# The current status of infrastructure for monitoring the efficacy of antimalarial therapeutics in Zambia

**DOI:** 10.5281/zenodo.10887816

**Published:** 2014-09-23

**Authors:** Ellah Zingani, Satoshi Inoue, Lungwani Tyson M. Muungo

**Affiliations:** 1 Department of Pharmacy, University of Zambia, School of Medicine, P.O. Box 50110, Lusaka, Zambia; 2 Department of Biological Science and Nursing, Yokohama City University, School of Medicine, 3-9 Fukuura, Kanazawa-ku, Yokohama 236-0004, Japan

## Abstract

**Background:**

Sub-Saharan countries have experienced centuries of high morbidity and mortality due to malaria. In addition to insecticide-treated mosquito nets and indoor residual spraying, modern antimalarial medicines have been developed to reduce disease prevalence, although the emergence of drug-resistant strains has compromised their efficacy. The purpose of this study was to evaluate the current status of malaria diagnosis and treatment, and to monitor the therapeutic efficacy of antimalarial drugs.

**Materials and Methods:**

A descriptive cross-sectional survey was conducted from 2011 to 2013 at 10 district hospitals in Zambia designated as malaria sentinel sites as well as at the National Malaria Control Centre. District medical officers at each site completed interview questionnaires.

**Results:**

Although basic infrastructure necessary for monitoring antimalarial drug resistance (such as laboratory, dispensary, admission ward, database unit, administration offices, bed space, examination and emergency rooms) was present at all sites, there was a shortage of licensed healthcare personnel. At some sites, antimalarial drugs were prescribed for malaria-like symptoms without diagnostic confirmation by blood smear. There was no regular monitoring of antimalarial drug resistance: only one trial was conducted among all sites in the previous 24 months.

**Conclusion:**

A lack of antimalarial drug resistance monitoring might be associated with personnel and funding shortages. Additional financial support would be necessary to avoid the development and spread of drug-resistant malaria in Zambia.

## 1 Introduction

Approximately 660,000 malaria-related deaths were reported worldwide in 2010 [1]. Vector control strategies, including insecticide-treated mosquito nets (ITNs) and indoor residual spraying (IRS), have dramatically reduced mortality and morbidity [1-3]. Effective antimalarial drugs have contributed to beneficial outcomes of disease control efforts. However, the emergence of drug resistance is of growing concern [1]. Concerted global efforts to combat malaria have been on-going since the 1950s [4]. Decreased incidence of disease was reported after development and deployment of chloroquine and sulfadoxine-pyrimethamine [5,6]. However, use of these drugs did not result in sustained disease control and they were no longer the most effective malaria treatments in tropical Africa by the early 2000s, mainly due to the spread of resistance to these drugs in most parts of the world [7-9]. Drug-resistant strains that emerge in local communities may be introduced to other regions and subsequently spread to other countries and eventually cause increased global malaria mortality [1,10]. Artemisinin, the active compound that is derived from the plant *Artemisia annua*, is currently one of the most effective antimalarial drugs. The World Health Organization (WHO) currently recommends artemisinin-based combination therapies (ACT) as a first-line therapeutic, although resistance to artemisinin has recently been reported in South Asia [1,11-13]. Chloroquine- and sulfadoxine-pyrimethamine-resistant strains have spread rapidly from Southeast Asia to Africa owing to increased global migration [6]. In sub-Saharan Africa, where antimalarial health care resources are limited [1,14], the consequences could be catastrophic if ACT efficacy would be reduced, particularly in rural areas. Prescription of antimalarial drugs may differ between sentinel sites because resistance patterns are heterogeneous even within limited geographic regions [4,15]. The spread of resistance might be avoided if appropriate treatments are selected in response to drug efficacy surveillance efforts. It is therefore important to study the geographical distribution of drug resistance in malaria-endemic countries [1,16].

The Republic of Zambia has experienced centuries of high mortality and morbidity from tropical infections such as malaria [3]. More than 4 million cases and 4,500 deaths due to malaria were reported in 2011, a large social and economic burden for communities [2]. The National Malaria Control Programme (1995–2000) tested antimalarial drug efficacy in several regions and found that chloroquine, which had been used regularly since the 1960s, was no longer an effective malaria treatment [14]. The second-line antimalarial drug used for chloroquine treatment failure in the late 1990s was sulfadoxine-pyrimethamine, although increasing resistance was observed [17]. In response to these findings, the national treatment policy was revised and ACT was adopted as a first-line treatment in 2002 [16,18]. After the introduction of Zambia’s National Malaria Control Strategic Plan in 2000, ITNs, IRS and rapid diagnostic tests were implemented in addition to ACT, resulting in a dramatic decline in malaria mortality, morbidity and hospital admission rates [1-3,16,18]. Effective systems for the regular monitoring of both malaria incidence and drug efficacy need to be maintained and updated to avoid resurgence of drug-resistant strains [19]. We conducted descriptive cross-sectional surveys in ten malaria sentinel sites to evaluate the current status of infrastructure for malaria treatment and monitoring of antimalarial drug therapeutic efficacy in Zambia.

**Table 1.** Staffing and facilities present at the ten District hospitals enrolled in the survey.

	Province	North-Western	Copper belt	Central	Lusaka	Eastern	Southern	Northern		Luapula	Western
	Population* (2010)	706,462	1,958,623	1,267,803	2,198,996	1,707,731	1,606,793	1,759,600		958,976	881,524
	Site	Mwinilunga	Chingola	Chibombo	Chongwe	Chipata	Kalomo	Isoka	Kaputa	Samfya	Senanga
Staffing	Doctor	3	6	2	3	15	3	1	1	1	3
	Nurse	26	80	16	11	88	78	52	16	34	36
	Pharmacist	2	2	0	3	3	2	1	0	0	0
	Lab Tech	1	4	3	3	8	2	2	2	2	2
	C Officer	2	8	7	5	19	9	1	1	2	1
Facilities	Laboratory	Y	Y	Y	Y	Y	Y	Y	Y	Y	Y
	Dispensary	Y	Y	Y	Y	Y	Y	Y	Y	Y	Y
	A Ward	Y	Y	Y	Y	Y	Y	Y	Y	Y	Y
	Exam Rm	Y	Y	Y	Y	Y	Y	Y	Y	Y	Y
	ER	N	Y	Y	Y	Y	Y	Y	Y	Y	N
	Stores	Y	Y	Y	Y	Y	Y	Y	Y	Y	Y
	Admin	Y	Y	Y	Y	Y	Y	Y	Y	Y	N
	Database	N	Y	Y	Y	Y	Y	Y	Y	Y	N
	Bed Cap	80	200	157	20	458	125	72	50	155	96

* Source: Central Statistical Office, Zambia.

Lab Tech = Laboratory Technician; C Officer = Clinical Officer; A Ward = Admission ward; Exam Rm = Examination room; ER = Emergency Room; Bed Cap = Bed Capacity; Y = yes; N = no.

## 2 Materials and Methods

### 2.1 Study site and design

A descriptive cross-sectional study was conducted. All malaria sentinel sites in Zambia were included in this study. These were the District hospitals in Chibombo, Chongwe, Chingola, Kalomo, Mwinilunga, Chipata, Isoka, Samfya, Senanga, and Kaputa, that were investigated to determine the infrastructure status for malaria prevention and treatment. Further information was obtained from the National Malaria Control Centre (NMCC). All investigated hospitals, classified as level 3 health institutions, were designated malaria sentinel sites for monitoring the efficacy of antimalarial drug therapeutics based on WHO protocols (not randomly selected). Sites were visited between July 2011 and February 2013. A questionnaire (appendix 1) was administered to the district medical officer in each hospital site. Face-to-face interviews were also conducted to confirm the information provided in questionnaires. Informed consent was obtained from each respondent prior to participation in the study.

### 2.2 Ethical consideration

This study received full approval from the University of Zambia Biomedical Research Ethics Committee. The study was assigned ethical reference number 13611.

## 3 Results

At all sites the basic equipment required for malaria treatment (laboratory, dispensary, wards, examination and emergency rooms, admission wards, stores, administration, database unit and patient bed space) was available, and monitoring of antimalarial drug efficacy was possible (Table 1). The average number of licensed medical staff per hospital was as follows (average ± s.d.): doctors=3.8±4.2; nurses 43.7±29.1 and pharmacists 1.3±1.3. The hospitals were responsible for providing professional medical services to a large number of patients regardless of malaria infection status: 45,736 ± 44,608 patients per year across 10 hospitals (Tables 1 and 2). There was no pharmacist at four of the sites (Chingola, Kaputa, Luapula and Senanga). In six of the 10 hospitals (Mwinilunga, Chingola, Kalomo, Isoka, Kaputa and Samfya), the number of patients who received diagnostic blood smears for malaria was less than the total number of antimalarial drug prescriptions. In Mwinilunga, for example, 266 patient blood smears were performed, but antimalarial drugs were prescribed to 38,151 patients (Table 2). The WHO protocol for monitoring drug efficacy was available in eight hospitals as well as in the NMCC (but not in Mwinilunga and Chipata) (Table 3). No medical staff were trained or involved in monitoring in any of the hospitals. At the NMCC, several medical staff appeared to be trained and involved in resistance monitoring, but specific information was not provided (at least five, supposed to be five staff members) (Table 3). In the previous 24 months, only one hospital (Chongwe) monitored antimalarial drug efficacy, and performed this testing only once (Table 3). A nationwide monitoring trial was conducted by the NMCC in 2006, but Kaputa was excluded owing to lack of funding. Only one of the ten hospitals (Isoka) monitored drug efficacy for tropical infections other than malaria (HIV and tuberculosis). No hospitals or the NMCC had adequate funding for testing.

**Table 2.** Number of patients, the number receiving malaria treatment, and the number of blood smear tests undertaken per District hospital.

Site	Patients/year	Patients treated for malaria/year	Number of blood smear tests (as % of patients treated for malaria/year)
Mwinilunga	126,987	38,151	266 (0.7)
Chingola	80,426	300	158 (52.6)
Chibombo	14,955	171	963 (563.2)
Chongwe	24,099	157	2,875 (1831.2)
Chipata	24,880	13,905	18,175 (130.7)
Kalomo	100,426	856	500 (58.4)
Isoka	8,000	3,200	500 (15.6)
Kaputa	unknown	1,350	322 (23.9)
Samfya	21,868	11,076	10,925 (98.6)
Senanga	9,982	526	1,810 (344.1)

## 4 Discussion

Although equipment was available, only one hospital monitored antimalarial drug therapeutic efficacy in the 24 months prior to this investigation. No medical staff was involved in or trained to monitor efficacy at any of the hospital sites. Nationwide trials had not been conducted since 2006 and the reported number of personnel trained to perform this monitoring was somewhat ambiguous. The delay in updating the status of drug efficacy might be due to inadequate financial resources [1,14]. The beneficial outcomes that were the result of interventions by the National Malaria Control Strategic Plan in 2000 have not been sustained. The WHO has reported malaria resurgence in some areas in Zambia; malaria deaths and cases among children under 5 years of age steadily declined until 2008, and several provinces reported malaria recurrence in 2009 and 2010 [2,14]. Consistent with our data, official reports on drug efficacy have not been released since 2006. Highly qualified and substantial data are required to revise the treatment policy [16]. There is an urgent need for an effective infrastructure for monitoring of drug resistance in Zambia to avoid recurrence of drug-resistant strains and a repeat of the unwanted healthcare situations observed in the 1990s.

There was a shortage of licensed personnel in all hospitals included in this study. In addition to malaria diagnosis and treatment, healthcare staff also provides medical services to non-malaria patients. Community healthcare workers (CHWs) trained to meet local healthcare needs are integral for reliable diagnosis and treatment of malaria [20,21]. The success of medical management by CHWs may depend on community acceptance of individual CHWs: this acceptance may change owing to diverse cultural values found in multi-tribe countries such as Zambia [22]. Although the situation would improve with an increased number of licensed medical staff, it is very difficult to retain highly-trained personnel owing to the drain of human resources from developing countries to members of the Organization for Economic Co-operation and Development (OECD): poor working conditions, higher crime rates and unstable political environment in developing countries as well as increased health care demand for aging populations in OECD countries means that hiring foreign-trained doctors and nurses from developing nations instead of training additional domestic medical personnel may be a more cost-effective solution for higher-income countries [23-25]. Better governance to improve working conditions for health workers in developing countries is therefore an urgent matter.

In six hospitals, more antimalarial drug prescriptions were prescribed than patients had been diagnosed with malaria by blood smear, suggesting that antimalarial drugs were administered on the basis of symptomatic diagnosis without true diagnostic confirmation. Inaccurate diagnosis combined with administration of antimalarial medications could contribute to the emergence of drug-resistant malaria strains [1,2,14,26-28]. Additionally, malaria misdiagnosis in patients with non-malaria infections, such as HIV and tuberculosis, could lead to fatal outcomes [3,25]. ACT is a new option for treatment of resistant strains that has replaced traditional low-cost drugs in endemic areas. ACT use in these areas could have negative effects on cost-efficiency unless the drug is administered only to patients whose malaria infections are correctly diagnosed [1]. To overcome these ongoing crises, appropriate malaria diagnostic training for all hospital healthcare personnel is necessary.

**Table 3.** Frequency, dates, and follow-up of monitoring of antimalarial drug efficacy in all the hospitals including the NMCC.

Site	Mwini- lunga	Chingola	Chi- bombo	Chong- we	Chipata	Kalo- mo	Isoka	Kaputa	Sam- fya	Se- nanga	NMCC-comment
Protocol available	N	Y	Y	Y	N	Y	Y	Y	Y	Y	Y
Staff involved in monitoring trials	0	0	0	0	0	0	0	0	0	0	At least 5
Staff trained for monitoring trials	0	0	0	0	0	0	0	0	0	0	Supposed to be 5
Staff sent for training in AMDTMT*/yr	0	0	0	0	0	0	0	0	0	0	Supposed to be 5
	Frequency of monitoring trials in past 24 months										
Never	**×**	**×**	**×**	**×**			**×**	**×**	**×**	**×**	**×**
N/A					**×**	**×**					
	Date of last monitoring trial										2006 CWT** except Kaputa
24 months ago				**×**							
Unknown	**×**	**×**	**×**				**×**		**×**	**×**	
N/A					**×**	**×**		**×**			
	Follow-up after a monitoring trial										
Staff at site							**×**				**×**
Others (Govt, NGO)							**×**				
No follow-up	**×**	**×**	**×**	**×**	**×**	**×**		**×**	**×**	**×**	

* AMDTMT= Anti-malarial drug therapeutic monitoring test; ** CWT= Countrywide trials.

## 5 Limitations

The questionnaire used in this study was not pretested due to funding shortages; however, we have successfully identified critical issues in monitoring antimalarial drug therapeutic efficacy in Zambia. The questionnaire is going to be updated for further epidemiology studies to improve antimalarial drug therapy.

## 6 Conclusions

The infrastructure developed in the 2000s to monitor the efficacy of antimalarial drug efficacy in Zambia contributed to highly beneficial outcomes; however, this infrastructure has been disrupted owing to a shortage of both experienced personnel and funding. Malaria isolates resistant to current antimalarial drugs have been identified in Southeast Asia, making the need for additional financial support more urgent for continued improvement in lowering the malaria burden in African nations such as Zambia.
